# Accelerating 3D MTC-BOOST in patients with congenital heart disease using a joint multi-scale variational neural network reconstruction

**DOI:** 10.1016/j.mri.2022.06.012

**Published:** 2022-10

**Authors:** Anastasia Fotaki, Niccolo Fuin, Giovanna Nordio, Carlos Velasco Jimeno, Haikun Qi, Yaso Emmanuel, Kuberan Pushparajah, René M. Botnar, Claudia Prieto

**Affiliations:** a Department of Biomedical Engineering, School of Biomedical Engineering and Imaging Sciences, King's College London, London, United Kingdom; b Guy's and St Thomas' NHS Foundation Trust, London, UK; c Escuela de Ingeniería, Pontificia Universidad Católica de Chile, Santiago, Chile

**Keywords:** Cardiac MRI, Neural network, Free-breathing, 3D whole-heart imaging, BART, Berkeley Advanced Reconstruction *Toolbox*, bSSFP, balanced Steady-State Free Precession, CHD, Congenital Heart Disease, CI, Confidence Interval, CS, Compressed Sensing, DNN, Deep Neural Networks, jMS-VNN, joint Multi-Scale Variational Neural Network, MTC-BOOST, Magnetization Transfer Contrast Bright blOOd phase SensiTive, PSIR, Phase Sensitive Inversion Recovery, RL, Right-Left, SI, Superior-Inferior, SM, Supplementary Material, SNR, Signal to Noise Ratio, T2prep-3DWH, T2-prepared 3D whole-heart, VD-CASPR, Variable-Density CArtesian Spiral PRofile order, VNN, Variational Neural Network, 3D-WH, 3D Whole-heart, 2D, 2 Dimensional, 3D, 3 Dimensional

## Abstract

**Purpose:**

Free-breathing Magnetization Transfer Contrast Bright blOOd phase SensiTive (MTC-BOOST) is a prototype balanced-Steady-State Free Precession sequence for 3D whole-heart imaging, that employs the endogenous magnetisation transfer contrast mechanism. This achieves reduction of flow and off-resonance artefacts, that often arise with the clinical T2prepared balanced-Steady-State Free Precession sequence, enabling high quality, contrast-agent free imaging of the thoracic cardiovascular anatomy. Fully-sampled MTC-BOOST acquisition requires long scan times (~10–24 min) and therefore acceleration is needed to permit its clinical incorporation. The aim of this study is to enable and clinically validate the 5-fold accelerated MTC-BOOST acquisition with joint Multi-Scale Variational Neural Network (jMS-VNN) reconstruction.

**Methods:**

Thirty-six patients underwent free-breathing, 3D whole-heart imaging with the MTC-BOOST sequence, which is combined with variable density spiral-like Cartesian sampling and 2D image navigators for translational motion estimation. This sequence acquires two differently weighted bright-blood volumes in an interleaved fashion, which are then joined in a phase sensitive inversion recovery reconstruction to obtain a complementary fully co-registered black-blood volume. Data from eighteen patients were used for training, whereas data from the remaining eighteen patients were used for testing/evaluation. The proposed deep-learning based approach adopts a supervised multi-scale variational neural network for joint reconstruction of the two differently weighted bright-blood volumes acquired with the 5-fold accelerated MTC-BOOST. The two contrast images are stacked as different channels in the network to exploit the shared information. The proposed approach is compared to the fully-sampled MTC-BOOST and 5-fold undersampled MTC-BOOST acquisition with Compressed Sensing (CS) reconstruction in terms of scan/reconstruction time and bright-blood image quality. Comparison against conventional 2-fold undersampled T2-prepared 3D bright-blood whole-heart clinical sequence (T2prep-3DWH) is also included.

**Results:**

Acquisition time was 3.0 ± 1.0 min for the 5-fold accelerated MTC-BOOST versus 9.0 ± 1.1 min for the fully-sampled MTC-BOOST and 11.1 ± 2.6 min for the T2prep-3DWH (*p* < 0.001 and *p* < 0.001, respectively). Reconstruction time was significantly lower with the jMS-VNN method compared to CS (10 ± 0.5 min vs 20 ± 2 s, *p* < 0.001). Image quality was higher for the proposed 5-fold undersampled jMS-VNN versus conventional CS, comparable or higher to the corresponding T2prep-3DWH dataset and similar to the fully-sampled MTC-BOOST.

**Conclusion:**

The proposed 5-fold accelerated jMS-VNN MTC-BOOST framework provides efficient 3D whole-heart bright-blood imaging in fast acquisition and reconstruction time with concomitant reduction of flow and off-resonance artefacts, that are frequently encountered with the clinical sequence. Image quality of the cardiac anatomy and thoracic vasculature is comparable or superior to the clinical scan and 5-fold CS reconstruction in faster reconstruction time, promising potential clinical adoption.

## 1 Introduction

Congenital heart disease (CHD) affects approximately 8–10 in 1000 live births globally with ~90% of this patient group surviving into adulthood [1,2]. MRI plays a key role in the diagnosis, pre-procedural planning and follow-up of patients with CHD [3]. The T2-prepared, three-dimensional, whole-heart, balanced steady-state free precession (T2prep-3DWH) sequence is most commonly utilized for bright-blood anatomical evaluation of the intra-cardiac anatomy and thoracic vasculature [[3], [4], [5], [6]]. Known limitations of this method include off-resonance artefacts mostly noted in the pulmonary veins and flow-related artefacts in cases of turbulent flow due to valvar or vascular stenosis or regurgitation, which are common occurrences in this population [7,8]. Additionally, the diaphragmatic navigator gating leads to long and unpredictable scan time, as only a fraction of the acquired data (usually at end-expiration) is utilized for image reconstruction; typically scan efficiency ranges between 30 and 45% [9]. Therefore, the application of a sequence with a different contrast mechanism, that can obviate the aforementioned shortcomings and operate with predictable examination time would be of significant clinical merit.

A recently developed prototype sequence enables simultaneous 3D Bright and black blOOd phase SensiTive (BOOST) imaging under free breathing [10]. This sequence exploits magnetization transfer contrast (MTC) to ensure high quality depiction of both the arterial and the venous system without the need of contrast agent injection and has been preliminary evaluated in patients with atrial fibrillation providing high-quality 3D whole-heart bright-blood imaging. Thus, it holds promise for contrast-free delineation of the cardiovascular anatomy in adults with CHD, circumventing the impediments associated with the conventional approach. Additionally, image-based navigators are used to compensate for the translational respiratory-induced motion of the heart [11]. This technique achieves 100% respiratory scan efficiency in a predictable scan time. In spite of these developments, fully-sampled MTC-BOOST requires long scan times (~12 min for non-isotropic 1.4 × 1.4 × 2.8 mm^3^ resolution and would require ~24 min to obtain images with a 1.4 mm^3^ isotropic resolution). Thus, further developments are needed to permit its incorporation into an efficient clinical protocol.

Several undersampling reconstruction techniques have been proposed to speed up the acquisition of 3D whole-heart MRI, including parallel imaging [12,13] and compressed sensing (CS) reconstruction [14,15]. CS works under the assumption that the k-space data is randomly undersampled, the image has a sparse representation in some pre-defined basis or dictionary and a nonlinear reconstruction is performed to enforce the sparsity of the image and consistency with the acquired MR data. For CS-based variable splitting techniques, the optimization problem is usually solved using an iterative coordinate-descent algorithm, alternating between the regularization operator step and the data consistency step. Despite the high promise of CS approaches, in practice the sparsifying transforms employed in CS (e.g. total variation or wavelets operators), may be too simple to capture the complex image content associated with cardiac MRI images. This may lead to reconstructions that appear overly smooth or unrealistic. A further major drawback is the long computational time typically required for the iterative solution of the associated non-linear optimization problem and the need for tuning of reconstruction hyper-parameters.

Recently, deep neural networks (DNN) have been proposed to overcome these challenges by learning optimal reconstruction parameters and/or transforms from the data itself and enabling extremely fast computational times (after training), promising to further advance the field of undersampled/accelerated MRI reconstruction [16,17]. DNN has been proposed to learn operations that are similar to those performed in conventional CS iterative reconstruction. Instead of hand-engineering the sparsifying transform, DNN methods can directly learn this regularization term by using convolutional neural networks. These techniques, such as Deep-Alternating Direction Method of Multipliers net [18], VNN [19] or CascadeNet [20], represent a deep-learning framework of an unrolled version of the iterative constrained reconstruction where the network parameters are trained in order to recover the images directly from undersampled k-space data as an input. In the corresponding experiments, cardiac MRI training and validation data were produced by retrospective undersampling complex images obtained from single-coil data. Other techniques have applied an unrolled end-to-end framework in the more realistic scenario of multi-channel coil complex MR data. In particular, Hammernik et al. introduced a trainable formulation for accelerated parallel-imaging based MRI reconstruction [19], which embedded a parallel-imaging and a CS reconstruction within a deep-learning unrolled end-to-end framework. Undersampled k-space data and coil sensitivity maps were provided as input to this unrolled model for deep-learning reconstruction, and high-quality MR images are obtained as an output in an end-to-end fashion. The regularization term of this network was implemented as a Variational Neural Network (VNN), and the data consistency term was implemented as the L2 norm with respect to the acquired k-space data. A multi-scale VNN approach (MS-VNN) has recently been proposed for highly undersampled (~9×) free-breathing coronary MR angiography [20]. Building on this work, we propose a joint Multi-Scale Variational Neural Network (jMS-VNN) for the reconstruction of multi-contrast high-resolution bright- and black blood MTC-BOOST datasets.

The proposed jMS-VNN, in combination with a variable-density spiral-like cartesian trajectory [21], was applied to prospectively acquired multi-coil 5-fold undersampled data with a 1.4 × 1.4 × 2.8 resolution. The proposed approach enables the acquisition of high-resolution MTC-BOOST 3D whole-heart images in ~2–4 min, and their joint reconstruction in ~20s offering easy integration in the clinical workflow.

The proposed jMS-VNN technique is evaluated in a cohort of 18 CHD patients and compared with Compressed-Sensing (CS) reconstruction, fully-sampled images and the conventional T2prep-3DWH.

## 2 Methods

The framework presented hereafter jointly reconstructs accelerated multi-channel multi-contrast images from an MTC-BOOST acquisition. A description of the proposed acquisition framework is presented followed by the description of the joint Multi-Scale reconstruction Network.

### 2.1 Acquisition Framework

**Fig. 1:**
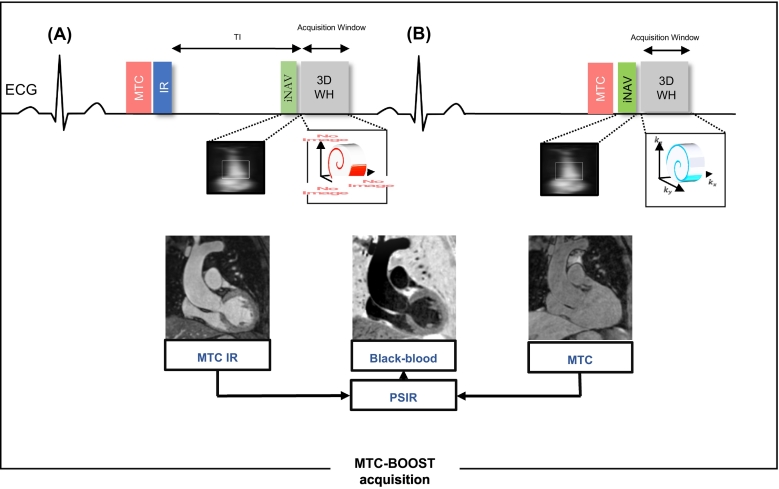
MTC-BOOST acquisition framework. Two magnetization prepared bright-blood volumes are acquired in odd and even heartbeats. Magnetization transfer in combination with an inversion pulse is used in odd heartbeats (A), whereas magnetization transfer solely is exploited in even heartbeats (B). In odd heartbeats, a short inversion time (TI), Inversion Recovery approach is used to suppress the signal from epicardial fat, whereas frequency-selective pre-saturation is used in even heartbeats. Data acquisition is performed using a 3D Cartesian trajectory with spiral profile order and segmented over multiple heartbeats (green, red, blue). A low-resolution 2D iNAV is acquired in each heartbeat by spatially encoding the ramp-up pulses of the bSSFP sequences. The bright-blood MTC-IR BOOST and MTC BOOST volumes are translational motion corrected at the end-expiratory level and, subsequently, combined in a PSIR-like reconstruction to generate a complementary black-blood volume. Abbreviations: bSSFP: balanced Steady-State Free Precession, MTC: Magnetization Transfer Contrast, IR: Inversion recovery pulse, PSIR: Phase sensitive inversion recovery. (For interpretation of the references to colour in this figure legend, the reader is referred to the web version of this article.)

A previously published ECG-triggered 3D Whole-heart bright-blood and black-blood Phase Sensitive Inversion Recovery (PSIR) bSSFP sequence was introduced for sharp delineation of both the thoracic arterial and venous systems [10]. This MTC-BOOST implementation alternates the acquisition of an IR pulse preceded by MT preparation (bright-blood MTC-IR BOOST, Fig. 1A), whereas MT preparation solely (bright-blood MTC BOOST, Fig. 1B) is applied at even heartbeats. In odd heartbeats, a short TI IR approach is used to suppress the signal from epicardial fat (Fig. 1A), whereas frequency-selective pre-saturation is used in even heartbeats (Fig. 1B).

A variable density spiral-like Cartesian (VD-CASPR) trajectory was utilized in this study to perform a 5-fold accelerated undersampled acquisition [21]. With this trajectory, the k_y_ − k_z_ phase-encoding plane was sampled following approximate spiral interleaves on the Cartesian grid with variable density along each spiral arm. The k_y_ − k_z_ plane was then segmented in two sets of concentric rings, the first defining the fully sampled k-space center and the second representing the accelerated spiral branches. The size of the fully sampled k-space center was set to 20% of the size of k_y_ and *k*_*z*_ encoding directions. As opposed to other undersampled trajectories such as radial or even uniform Cartesian trajectories where strong aliasing and streaking artefacts are usually observed, the proposed Cartesian variable-density ensures a pseudo-random pattern, resulting in incoherent aliasing that spreads irregularly in a noise-like fashion. This makes the proposed trajectory a good candidate for deep-learning based reconstruction, which is well-suited to suppress noise-like artefacts without degrading image quality.

### 2.2 Translational Motion Estimation

In each heartbeat, a low resolution 2D image-based navigator (iNAV) is acquired by spatially encoding the ramp-up pulses of the bSSFP sequence, therefore allowing for the estimation of respiratory motion along the superior-inferior (SI) and right-left directions (RL), and 100% scan efficiency [11]. Respiratory motion estimation and compensation is performed in a beat-to-beat 2D translational fashion for the 2 bright-blood data sets (MTC-IR BOOST and MTC BOOST) independently. The beat-to-beat translational respiratory motion of the heart is estimated along the SI and RL directions using a template-matching algorithm exploiting cross-correlation, with the template manually selected around the heart during acquisition planning.

### 2.3 Multi Contrast Image Reconstruction

#### 2.3.1 Problem Formulation

Let ***ρ*** ∈ *ℂ*^[*N*_*x*_,*N*_*y*_,*N*_*z*_,*L*]^ be the multi-contrast complex images that we seek to reconstruct, where *N*_*x*_, *N*_*y*_ *and N*_*z*_ are the number of voxels in the *x*, *y and z* spatial directions, and *L* = 2 is the number of contrast-weighted images. The corresponding complex receive-coil sensitivity maps for the Nc channels are denoted as *S* ∈ *ℂ*^[*N*_*x*_,*N*_*y*_,*N*_*z*_,*N*_*c*_,*L*]^. Let ***κ*** ∈ *ℂ*^[*K*,*L*,*N*_*c*_]^ be the undersampled k-space data (with *K* ≪ [*N*_*x*_, *N*_*y*_, *N*_*z*_]). The joint multi-contrast undersampled reconstruction can be combined with parallel imaging and cast the following inverse problem:

(1) $$\underset{\rho }{\text{argmin}}\left\{\frac{\lambda }{2}{\left\|\mathrm{AFS\rho}-k\right\|}_{2}^{2}\right\}$$

where A is the undersampling operator that acquires k-space data for each contrast-weighted image, F denotes the Fourier transform operator and ‖ · ‖ is the Frobenius norm.

Mathematically, this inverse problem is ill-posed, in the sense that the exact solution might not exist or not be unique, making precise recovery of ***ρ*** difficult. Prior assumptions on the unknown solution ***ρ*** have to be considered and the reconstruction problem can therefore be resolved applying the following constrained optimization framework:

(2) $$\underset{\rho }{\text{argmin}}\left\{\frac{\lambda }{2}{\left\|\mathrm{AFS\rho}-k\right\|}_{2}^{2}+\lambda P\left(\rho \right)\right\}$$

The first term in Eq. (2) enforces data consistency to the translational motion-compensated undersampled k-space data ***k***. The second term represents a regularization operator on ***ρ*** and *λ* is the hyper-parameter that adjust the influence of the data fidelity term based on the noise level of the acquired measurements **k**.

In CS-based reconstruction methods, the optimization problem in Eq. (2) is usually solved using an iterative algorithm that alternates between the regularization operator step and the data consistency step. A variety of pre-defined regularization operator$\;P\left(\rho \right)$ can be chosen e.g. total variation, spatial wavelets. However, the sparsifying transforms employed in CS may be too simple to capture the complex image content associated with cardiac MRI images, and lead to reconstructions that appear unnatural. Instead of hand-engineering the regularization term, several deep-learning methods have been proposed to directly learn the regularization term by using convolutional neural networks. In this work, we propose a joint Multi-Contrast VNN implementation for the reconstruction of 3D bright and black-blood cardiac MRI volumes from 2D multi-coil axial data.

#### 2.3.2 A Joint Multi Scale variational Network for Multi Contrast Reconstruction

**Fig. 2:**
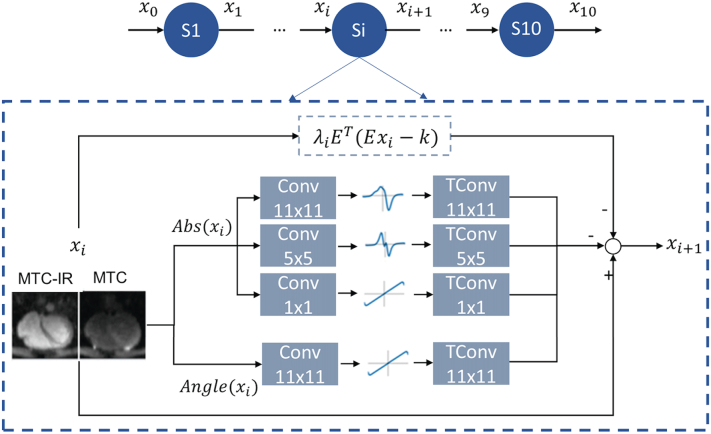
Joint multi-scale variational neural network architecture. Network architecture of the joint multi-scale VNN (jMS-VNN), which consists of 10 stages. In each stage, the first path enforces data consistency, whereas the second path is the regularization operator that applies convolutional filters (Conv), learnable activation functions and corresponding transposed convolutional filters (TConv) respectively to the magnitude and phase of the complex-valued MTC-IR and MTC images. For the magnitude image, a multi-scale approach is applied with three parallel filter sets with size of 11 × 11, 5 × 5 and 1 × 1. For the phase image, one set of 11 × 11 filter kernels is applied. The MTC-IR and MTC images are stacked as different channels for the network input to exploit their shared information. Abbreviations: jMS-VNN: joint multi-scale variational neural network, MTC: Magnetization transfer contrast, IR: Inversion recovery pulse, TConv: transposed convolutional filters.

The architecture of the proposed MS-VNN is depicted in Fig. 2. It is based on a previously proposed MS-VNN approach [20]. Translational motion corrected multi-coil undersampled 2D k-space MTC-IR and MTC bright-blood data and corresponding coil sensitivity maps are stacked in the channel dimension and provided as input to an unrolled model for deep-learning reconstruction. The proposed unrolled MS-VNN architecture consists of 10 network stages and each stage corresponds to a gradient update of a classic iterative reconstruction approach as in Eq. (2). The regularization term of this network was implemented as a joint Multi Scale Variational Neural Network, and the data consistency term was implemented as the L_2_ norm with respect to the acquired k-space data.

The joint multi-scale regularization term $P^{2D}$ for a real-valued 2D axial image ***ρ***_*j*_ is here defined as a Fields of Experts model:

(3) $$P^{2D}\left([\rho_{j}\right)=\sum_{s=\left[1511\right]}\sum_{f=1}^{\mathrm{FK}}\langle \Phi_{f,s}\left(\chi_{f,s}\rho_{j}\right),1\rangle$$

where $P^{2D}\;$is a linear operator that models convolutions of the 2D axial image ***ρ***_*j*_ with *f* = 1…*FK* filter kernels χ_f, s_ ∈ *ℝ*^*s*×*s*^ of size *s*, and the real-valued non-linear potential function Φ_*f*, *s*_. In the Fields of Experts model, both convolution kernels and parametrization of the non-linear potential functions are learned from the data, shifting the key effort of optimization from the online reconstruction stage to an up-front offline training task.

The proposed MS-VNN learns two separate sets of kernels and activation functions for the magnitude and phase images of the complex-valued data. The joint multi-scale regularization term is therefore applied separately to magnitude and phase as in equation:

(4) $$P^{2D}\left(\rho_{j}^{t}\right)=\sum_{f=1}^{\mathrm{FK}}\left(\sum_{s=1,5,11}P_{s}^{2D}\left(\mathrm{abs}\left(\rho_{j}^{t}\right)\right)\right)\;\exp\left(i*P_{s=11}^{2D}\left(\mathrm{angle}\left(\rho_{j}^{t}\right)\right)\right)\;$$

For the magnitude component, three sets of filters (FK_MAG_) and activation functions (AF_MAG_) were learned in parallel: FK_MAG1_ = 24 filters of size 11 × 11, FK_MAG2_ = 12 filters of size 5 × 5 and FK_MAG3_ = 6 filters of size 1 × 1. Their corresponding activation functions were each defined by AF_MAG_ = 31 RBFs. For the phase component, FK_PH_ = 12 filters of size 11 × 11 were learned. The corresponding activation function were defined by AF_PH_ = 31 RBFs. The phase path has one scale of representation due to the smooth nature of the phase images. Separating the learning for the magnitude and the phase image permits to apply the multi-scale approach only to the magnitude image with much less computational effort, less training time, and no substantial difference in image quality.

In order to perform a joint reconstruction of MTC-IR and MTC bright-blood data, the parameters of the network are shared among the two network channels. The previously proposed MS-VNN approach [20], has been extended for the joint reconstruction of bright- and black blood images. A manual hyperparameter search was conducted using the configuration that performed best on our training data. The selection of network configuration/hyperparameters of the jMS-VNN was performed heuristically, based on previous experience with the foundation MS-VNN network.

At the last stage of the network, the output MTC-IR BOOST and MTC-BOOST images are combined in a PSIR-like reconstruction as described in Kellman et al. [22], generating a third, complementary, PSIR MTC-BOOST image.

#### 2.3.3 Training

All parameters of this formulation, including the prior model defined by filter kernels, activation functions and data term weights, were learned during an off-line training procedure. The network was trained on 2D axial images obtained from 18 patients by retrospectively undersampling to 5-fold acceleration the fully-sampled 3D motion-compensated MTC-IR and MTC data (resulting in N_x_×18 × 2 = 8760 2D axial images). 8000 randomly selected axial images were used for training and 760 axial images were used for validation. The training set was split into mini batches of size 40. The 2D bright blood MTC-BOOST and the black-blood PSIR MTC-BOOST image outputs of the MS-VNN were compared to the corresponding fully sampled reference axial images during training. Optimization was performed for 800 epochs with a step size of η = 10^−3^, using the iPalm algorithm [23]. Training and validation were performed with retrospective undersampling data to ensure that the effect of respiratory and cardiac motion in both output and target images was identical. After training, jMS-VNN was used to reconstruct the prospectively 5-fold accelerated MTC-BOOST acquisitions of the remaining 18 CHD patients (not used in training).

### 2.4 Data Acquisition

MTC-BOOST was acquired on 36 patients with CHD on a 1.5 T system (MAGNETOM Aera, Siemens Healthcare). Patients were prospectively enrolled for the study from June 2019 to September 2019. Further inclusion criteria were: (1) >18 years of age and (2) clinically indicated MRI scan. General MRI exclusion criteria were applied to patient selection. Patients' demographics were obtained by medical record chart review. Written informed consent was obtained from all patients, before undergoing the scans, and the study was approved by the National Research Ethics Service.

Imaging parameters are presented in Table 1.

**Table 1:** Imaging parameters for the clinical T2-prepared 3D whole-heart b-SSFP and the MTC-BOOST sequence.

Parameter	Clinical	Research
Sequence	T2prep-bSSFP	MTC-BOOST
Field strength	1.5 T	1.5 T
Coil type	18-channel chest coil & 32-channel spinal coil	18-channel chest coil & 32-channel spinal coil
Echo Time (TE)/Repetition Time(TR)	1.7/3.5 ms	1.5/3.2 ms
Cardiac gating	ECG-triggering	ECG-triggering
	mid-diastolic acquisition (90–130 ms)	mid-diastolic acquisition (90-130 ms)
Flip angle (degrees)	90°	Not applicable
Spatial resolution	1.4 mm^3^	1.4 × 1.4 × 2.8 mm (reconstructed to 1.4mm^3^ isotropic)
Acceleration	GeneRalised Autocalibrating Partial Parallel Acquisition (GRAPPA) 2-fold acceleration	Fully-sampled (& 5-fold acceleration in 18/36 patients)
Respiratory motion compensation	Diaphragmatic navigator (gating window of ±3.5 mm in end-expiration)	Image-based navigator
T2-prepation duration	40 ms	Not applicable
MT-preparation	Not applicable	15 Gaussian pulses, flip-angle 800^o^, frequency offset:3000 Hz, duration:20.5 ms

Abbreviations: b-SSFP: balanced-Steady State Free Precession, MT: magnetisation transfer, MTC-BOOST: Magnetisation Transfer Contrast Bright and black Blood phase SensiTive.

Two different acquisition strategies were followed. The first eighteen patients (cohort A) constituted the training patient cohort and were scanned with two sequences: 1) the clinical T2prep-3DWH and 2) the fully-sampled MTC-BOOST. The fully-sampled MTC-BOOST data were retrospectively undersampled with a factor of 5-fold (offline simulation) and used for the training of the network, so that both undersampled and fully-sampled images are reconstructed from exactly the same acquisition (therefore undergoing exactly the same cardiac and respiratory motion). The remaining 18 patients (cohort B), whose data was used for the validation of the network were scanned with three sequences: 1) the clinical T2prep-3DWH, 2) the 5-fold (5× prospectively undersampled inline) MTC-BOOST and 3) the fully-sampled MTC-BOOST. In this case the corresponding fully-sampled images were acquired after the undersampled scan, thus cardiac and respiratory motion may differ between both acquisitions.

### 2.5 Input Data Pre-processing

Before reconstruction, the acquired k-space data underwent the following pre-processing steps:

1) Respiratory motion correction was performed toward end-expiration by applying a linear phase shift in k-space, as previously described in Henningsson et al. [11].

2) Coil sensitivity maps were estimated using the method presented in Walsh et al. [24] from low resolution images obtained from the fully sampled k-space center.

3) Since we aim to reconstruct a 3D volume ***ρ*** from 2D axial slices, an FFT is applied along the motion-compensated k_x_ readout dimension of k-space data to enable training and reconstruction for each 2D plane of the 3D volume, termed ρ_j_ (with *j* = 1…*N*_*x*_ and *N*_*x*_ the number of samples in the readout direction), from the corresponding measurement k_j_ (with *j* = 1…*M*_*x*_ = *N*_*x*_). This pre-processing step permits straightforward parallelization of the training and reconstruction process. The advantage of training in 2D with respect to 3D is the increased number of the training data set for improved generalization.

The pre-processing steps 1) and 2) were also applied for the fully-sampled and 5-fold CS undersampled reconstructions.

### 2.6 Evaluation Experiments in Prospectively Undersampled Data

Prospectively undersampled MTC-BOOST data from 18 patients (not included in the training, cohort B), were used to evaluate the proposed framework. The acquired data were reconstructed by applying the proposed jMS-VNN technique and, for comparison, were also reconstructed with CS. CS reconstruction with L1-Wavelet regularization was employed, as implemented in the Berkeley Advanced Reconstruction Toolbox (BART) toolbox [25]. The CS regularization parameter was carefully tuned in a subset of images and set to *λ*_*cs*_ = 0.01 for all reconstructions. The CS algorithm was stopped after 10 iterations, because preliminary testing revealed that this number of iterations was sufficient to reach a convergent solution. All reconstructions were performed offline on a workstation with a 16-core Dual Intel Xeon Processor (2.3 GHz, 256 RAM) and a Nvidia Titan V GPU (12 GB memory, 640 Tensor Cores, 110 TeraFLOPS).

### 2.7 Visual Analysis

Analysis was performed for all subjects from cohort B for the proposed 5-fold jMS-VNN, the 5-fold CS, the fully-sampled MTC-BOOST and the reference T2prep-3DWH datasets. Two cardiologists (Y.E. & K.P., 15 and 10 years of experience, respectively) graded the image quality of bright-blood images. In total, 72 sets were randomized and de-identified for display on a 3D workstation (Osirix v.9.0, OsiriX Foundation, Geneva, Switzerland). Before visual evaluation, the two readers were given training data sets with poor to excellent quality to calibrate their scores together. Following this training session, each reader was blinded to image acquisition type, reconstruction method, the other reader, and clinical history for independent evaluation. Diagnostic quality of the coronary arteries, pulmonary veins, great arteries, neck vessels and the superior vena cava was assessed using a 4-point Likert scale (1: non-diagnostic to excellent: 4 image quality).

### 2.8 Quantitative Bright-Blood Image Quality Analysis

Quantitative evaluation of the bright-blood image quality for the respective structures was performed by a third cardiologist (AF, 3 years of experience, Level III cardiac MRI accreditation) by estimating the Signal to Noise ratio (SNR) for the intrapericardiac vascular structures. SNR is defined as:

$$\mathrm{SNR}=\frac{\mathrm{Mean Signal}\;\left(\mathrm{Region of Interest}\right)}{\mathrm{Standard Deviation of the Noise}\left(\mathrm{Image background},\mathrm{outside the chest}\;\right)}.$$

Furthermore, image quality comparison was performed using peak signal-to-noise ratio (PSNR) and structural similarity (SSIM) over the region of interest around the heart and the thoracic vessels of the 5-fold accelerated CS and 5-fold accelerated jMS-VNN. Fully-sampled images were used as reference in both cases.

### 2.9 Aortic Diameter Measurements

The third reader (A.F., 3  years of experience, Level III cardiac MRI accreditation) measured two diameters (longest and its orthogonal) at three standardized locations established by guidelines (1: sinotubular junction; 2: mid ascending aorta; 3: main pulmonary artery) on a 3D workstation. Anatomic landmarks were used to reformat the bright-blood volumes and find appropriate views.

### 2.10 Statistical Analysis

A Kolmogorov-Smirnov test was performed to test the null hypothesis that each variable is normally distributed at the 5% significance level. A paired *t*-test was performed for normal distribution and Mann-Whitney test for non-normal distribution. For statistical analysis between reconstruction methods, the two readers' scores were averaged. Establishing non-normal distribution, the Kruskal was used to compare the individual visual scores and signal to noise ratio of the structures between the methods.

The Bland-Altman analyses were conducted on vessel diameters to determine their agreement. A *p* value <0.05 was considered significant for all statistical tests.

## 3 Results

Free-breathing motion-compensated 3D whole-heart acquisitions and reconstructions were completed successfully in all subjects (*N* = 36, age: 32 ± 13 years; 20 male). Table 2 presents the testing patient cohort (*N* = 18, age: 37 ± 14 years; 11 male subjects, cohort B) with the corresponding cardiac diagnoses, including patients with obstructive right and left heart disease, single ventricle physiology, anomalous pulmonary venous drainage, stent implantation in the superior vena cava and the descending aorta, bioprosthetic valves and atrial flutter. The training patient cohort (cohort A) is shown in Supplementary Material Table 1 with corresponding diagnoses.

**Table 2:** Patient identification number (ID), age, corresponding diagnosis and procedures and patients' characteristics for the participants included in the testing of the network and analysis of the results (cohort B).

Patient ID	Age (years)	Diagnosis	Procedures
1	27	Bicuspid aortic valve	1. Balloon aortic valvuloplasty
			2. Ross procedure. Pulmonary homograft in situ.
2	31	Aortic stenosis	Ross procedure. Pulmonary homograft in situ
3	54	Aortic root dilatation and dilatation of the ascending aorta	None
4	27	Tricuspid atresia	1. Pulmonary artery banding
		Transposition of the great arteries	2. Fontan procedure
		Ventricular septal defect	
5	26	Bicuspid aortic valve	None
6	36	Transposition of the great arteries	Senning procedure
7	61	Pulmonary stenosis	None
8	18	Tetralogy of Fallot	1. Blalock Taussig shunt
			2. Tetralogy of Fallot repair
9	29	Pulmonary stenosis	Balloon valvuloplasty
10	30	Double chamber right ventricle	Double chamber right ventricle and ventricular septal defect repair
		Ventricular septal defect	
		Bicuspid aortic valve	
11	21	Coarctation of the aorta	Coarctation repair
		Bicuspid aortic valve	
12	28	Partial anomalous pulmonary venous drainage with right upper pulmonary vein to superior vena cava	None
13	58	Aortopulmonary window	Aortopulmonary window repair
		Atrial flutter	
14	55	Pulmonary hypertension workup	
15	50	Sinus venosus defect	Catheter closure of sinus venosus defect. Covered stent in situ.
16	24	Sinus venosus defect	Catheter closure of sinus venosus defect. Covered stent in situ.
17	36	Subaortic stenosis	1. Ventricular septal defect repair and re-suspension of aortic valve.
		Ventricular septal defect with aortic valve prolapse	
			2. Ross procedure.
			Pulmonary homograft in situ.
18	56	Coarctation of the aorta	1. Aortoplasty with Dakron patch
			2. Stent for re-coarctation

The 5-fold undersampled MTC-BOOST acquisition had a mean duration of 3 min (±1 min). The scan time for the fully-sampled MTC-BOOST acquisition was in average 9 min (±1.1), *p* < 0.001 whereas the T2prep-3DWH acquisition took 11.1 min (±2.6), *p* < 0.001. The scan time for the fully-sampled MTC-BOOST acquisition was also significantly shorter than the T2prep-3DWH sequence (*p* = 0.002). The reconstruction time for the CS was 10 min(±0.5) versus 20 s(±2 s) (*p* < 0.001) for the proposed jMS-VNN.

### 3.1 Visual Comparison for Bright and Black Blood MTC BOOST jMS-VNN Images

**Fig. 3:**
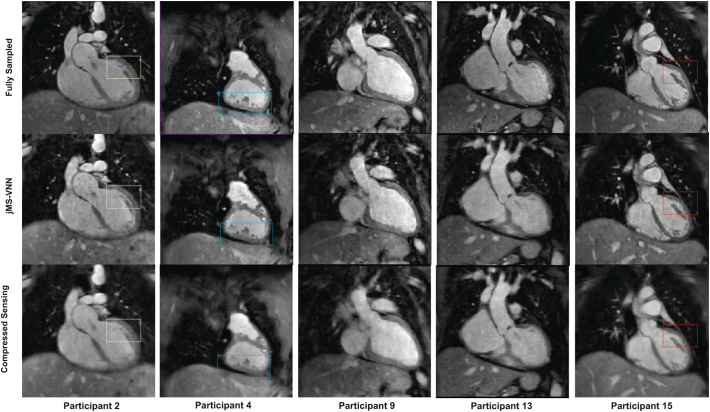
Fully sampled MTC-BOOST versus 5-fold jMS-VNN and 5-fold CS. Bright-blood images in coronal view for five representative participants. Acquisitions were performed with the fully-sampled MTC-BOOST sequence and 5-fold prospectively undersampled MTC-BOOST sequence. Accelerated MTC-BOOST reconstructed with Compressed Sensing (CS) introduced mild blurring that is mitigated with the jMS-VNN reconstruction (participant 2,4,15). Left ventricular wall and papillary muscles (yellow box), right ventricular trabeculations (blue box) and left ventricular wall and papillary muscles (red box) were sharper delineated with the jMS-VNN reconstruction in comparison to CS (participant 2,4,15 respectively). jMS-VNN showed similar image quality to the fully-sampled scan. Abbreviations: CHD: Congenital Heart Disease, CS: Compressed Sensing, jMS-VNN: Joint Multi Scale Variational Neural Network, MTC-BOOST: Magnetisation Transfer Contrast Bright and black blOOd phase SensiTive. (For interpretation of the references to colour in this figure legend, the reader is referred to the web version of this article.)

**Fig. 4:**
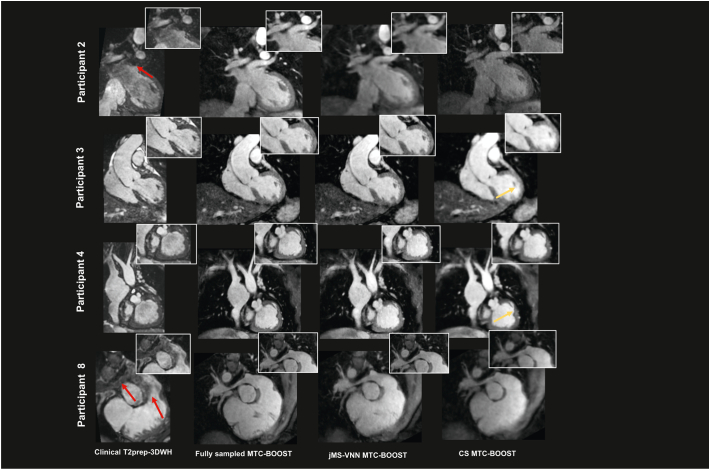
Clinical dataset versus fully sampled MTC-BOOST, 5-fold jMS-VNN and 5-fold CS. Bright blood images for four participants. Acquisitions were performed with the clinical sequence (T2 prepared 3D Whole-Heart), the fully-sampled MTC-BOOST sequence and 5× prospectively undersampled MTC-BOOST sequence. Accelerated MTC-BOOST was reconstructed with Compressed Sensing (CS) and joint Multi-Scale Variational Neural Network (jMS-VNN). Close-up views are shown for each reconstruction. MTC BOOST introduced uniform signal of the cardiac chambers and vessels in comparison to the clinical sequence, suppressing flow and off-resonance artefacts (participant 2 and 8, red arrows), that was preserved with both reconstruction methods, albeit with less noise artefacts in the jMS-VNN reconstruction. Residual blurring in the left and right ventricular wall (yellow arrow) was observed with the CS reconstruction that was reduced with the jMS-VNN. Abbreviations: CHD: Congenital Heart Disease, CS: Compressed Sensing, jMS-VNN: Joint Multi Scale Variational Neural Network, MTC-BOOST: Magnetisation Transfer Contrast Bright and black blOOd phase SensiTive, T2prep-3DWH: T2 prepared 3D Whole-Heart. (For interpretation of the references to colour in this figure legend, the reader is referred to the web version of this article.)

**Fig. 5:**
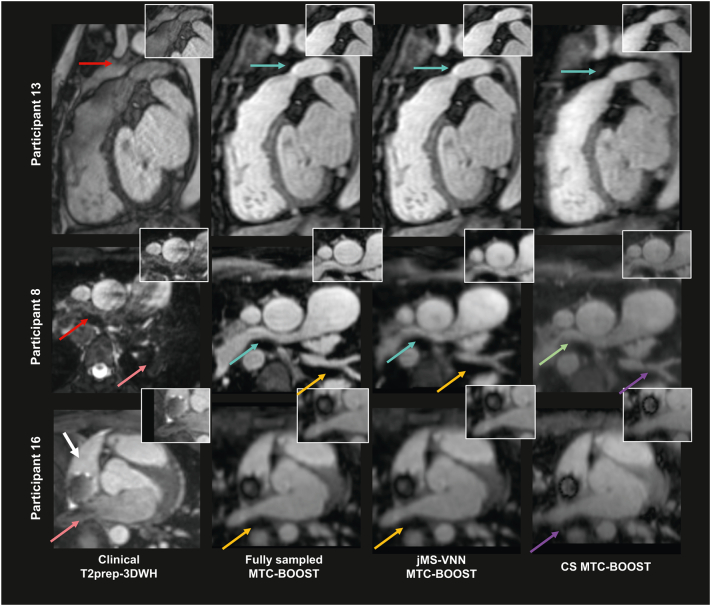
Clinical datasets with T2-preparation related MRI artefacts versus the corresponding fully sampled MTC-BOOST, 5-fold jMS-VNN and 5-fold CS. Significant flow related artefacts (red arrow) in the right ventricular outflow tract and main pulmonary artery in participant 13 and in the right pulmonary artery (red arrow) in participant 8, rendering the structures almost indistinguishable with the T2prep-3DWH sequence. Those were mitigated with the fully-sampled MTC-BOOST, 5-fold jMS-VNN and 5-fold CS (light blue and green arrows). Residual blurring was noted in the right pulmonary artery with the CS reconstruction in participant 8. Off-resonance artefacts in the left lower pulmonary veins and right upper pulmonary vein degraded significantly the image quality in the clinical sequence (participant 8 and 16 respectively, pink arrow). Those were attenuated with the fully-sampled MTC-BOOST and 5-fold jMS-VNN sequence (orange arrows). 5× CS introduced mild blurring (purple arrows). Artefact from the stent in the SVC was present in the clinical (white arrow) and the MTC-BOOST acquisition, however the vascular luminal signal and the pulmonary venous return was better appreciated in the fully-sampled MTC-BOOST sequence as well as in the 5-fold jMS-VNN and 5-fold CS. Abbreviations: CHD: Congenital Heart Disease, CS: Compressed Sensing, jMS-VNN: Joint Multi Scale Variational Neural Network, MTC-BOOST: Magnetisation Transfer Contrast Bright and black blOOd phase SensiTive, T2prep-3DWH: T2 prepared 3D Whole-Heart. (For interpretation of the references to colour in this figure legend, the reader is referred to the web version of this article.)

The 5-fold undersampled bright blood MTC-BOOST datasets reconstructed with the proposed jMS-VNN technique compared to the fully-sampled MTC-BOOST and to 5-fold undersampled with CS reconstruction are presented for representative participants (cohort B) in Fig. 3, Fig. 4, Fig. 5 (bright-blood) and SM Fig. 1 (black-blood). jMS-VNN reconstruction presented negligible differences to the fully-sampled in terms of image quality and reduced blurring and noise-like artefacts that were introduced with the CS reconstruction.

Fig. 5 additionally demonstrated attenuation of flow- and off-resonance artefacts with the proposed bright-blood MTC-BOOST, in comparison to the clinical sequence. This quality was preserved in the 5-fold reconstruction with jMS-VNN and CS, albeit with residual blurring in the CS. Artefact from the stent in the SVC was present in the clinical and MTC-BOOST acquisition, however the vascular luminal signal and the pulmonary venous return was better appreciated in the MTC-BOOST sequence and the 5-fold jMS-VNN and 5-fold CS.

### 3.2 Image Quality Scores Comparison for Bright-Blood MTC-BOOST jMS-VNN

**Fig. 6:**
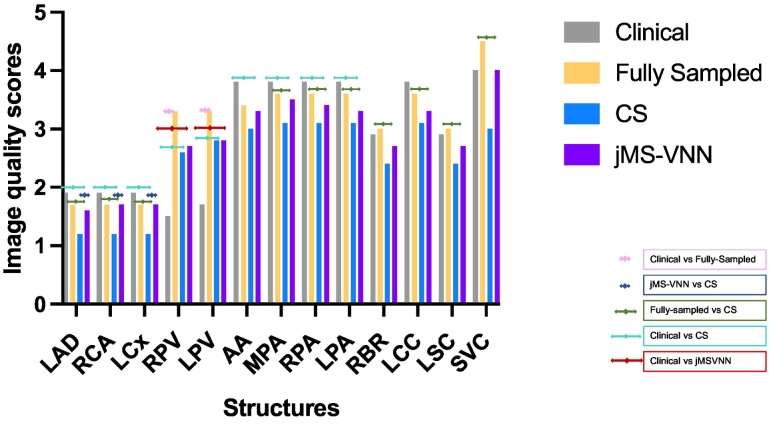
Image quality scores for the proposed 5-fold jMS-VNN MTC-BOOST in comparison to the clinical sequence, fully-sampled MTC-BOOST and 5-fold MTC-BOOST with CS reconstruction. Image quality scores for the proposed 5-fold jMS-VNN MTC-BOOST in comparison to the clinical sequence, fully-sampled MTC-BOOST and 5-fold MTC-BOOST with CS reconstruction for the two reviewers. Results have been averaged between the two reviewers. Abbreviations: AA: ascending aorta, CS: Compressed Sensing, jMS-VNN: Joint Multi Scale Variational Neural Network, LAD: left anterior descending coronary artery, LCC: left common carotid, LCx: left circumflex coronary artery, LPA: left pulmonary artery, LPV: left pulmonary vein, LSC: left subclavian, MTC-BOOST: Magnetisation Transfer Contrast Bright and black blOOd phase SensiTive, MPA: main pulmonary artery, RBR: right brachiocephalic, RCA: right coronary artery, RPA: right pulmonary artery, RPV: right pulmonary vein, SVC: superior vena cava.

Image quality scores averaged across both reviewers for the proposed 5-fold jMS-VNN MTC-BOOST in comparison to the clinical sequence, fully-sampled MTC-BOOST and 5-fold MTC-BOOST with CS reconstruction are shown in Fig. 6. Scores comparing the performance of all methods against the jMS-VNN along with statistical values are shown in Table 3 and an inter-method comparison between the clinical, fully-sampled MTC-BOOST and 5-fold CS is presented in SM Table 2.

**Table 3:** Inter-method comparison of bright-blood image quality scores for vascular structures acquired with 5-fold undersampling with jMS-VNN reconstruction versus the clinical T2prep-bSSFP, MTC-BOOST fully sampled and 5-fold undersampled with CS reconstruction.

Structure	Clinical	MTC-BOOST Fully sampled	MTC-BOOST Compressed Sensing	MTC-BOOST jMS-VNN	Clinical vs MTC-BOOST jMS-VNN	MTC-BOOST Fully sampled vs MTC-BOOST jMS-VNN	MTC BOOST Compressed Sensing vs MTC-BOOST jMS-VNN
LAD R.1	2(2,2)	2(1,2)	1(1,1.3)	2(1, 2)	*p* = 0.5	*p* > 0.99	*p* = 0.03*
LAD R.2	4(3,4)	3.5(3,4)	2(2.3)	3(2,4)	*p* = 0.18	*p* > 0.99	*p* = 0.08
LCx R.1	2(2,2)	2(1, 2)	1(1,1.3)	2(1, 2)	*p* > 0.99	*p* > 0.99	*p* < 0.0001*
LCx R.2	4(3,4)	3.5(3,4)	2(2,3)	3(2,4)	*p* = 0.1	*p* = 0.97	*p* = 0.2
RCA R.1	2(2,2)	2(1, 2)	1(1,1.3)	2(1, 2)	*p* > 0.99	*p* > 0.99	*p* = 0.0006*
RCA R.2	4(3,4)	3(2,4)	2(2.3)	2.5(2,3.3)	*p* = 0.06	*p* > 0.99	*p* = 0.1
AA R.1	4(4,4)	4(3,4)	3(3,3)	3(3,4)	*p* = 0.08	*p* > 0.99	*p* = 0.9
AA R.2	4(3.8,4)	4(3,4)	3(3,3.5)	4(3,4)	*p* = 0.99	*p* > 0.99	*p* = 0.04*
RPA R.1	4(4,4)	4(3,4)	3(3,3)	3.5(3,4)	*p* = 0.09	*p* = 0.8	*p* = 0.3
RPA R.2	4(3.8,4)	4(3.8,4)	3(3,3)	3(3,4)	*p* > 0.99	*p* > 0.99	*p* = 0.09
LPA R.1	4(4,4)	4(3,4)	3(3,3)	3(3,4)	*p* = 0.1	*p* = 0.8	*p* > 0.99
LPA R.2	4(3.8,4)	4(3.8,4)	3(3,3)	4(3,4)	*p* > 0.99	*p* > 0.99	*p* = 0.09
MPA R.1	4(4,4)	4(3,4)	3(3,3)	3.5(3,4)	*p* = 0.1	*p* > 0.99	*p* = 0.2
MPA R.2	4(3.8,4)	4(3.8,4)	3(3,)	4(3,4)	*p* > 0.99	*p* > 0.99	*p* = 0.1
RPV R.1	1(1,2)	3.5(2.8,4)	3(2,3)	3(2,3)	*p* = 0.003*	*p* = 0.39	*p* > 0.99
RPV R.2	2(1.8,2.3)	4(3,4)	3(2,3)	3.5(2.75,4)	*p* = 0.0005*	*p* > 0.99	*p* = 0.9
LPV R.1	1(1,2.3)	3.5(2.8,4)	3(2,3)	3(2.8,3)	*p* = 0.01*	*p* = 0.9	*p* > 0.99
LPV R.2	3(2,3.3)	3.5(3,4)	4(3,4)	3.5(3,4)	*p* = 0.09	*p* > 0.99	*p* = 0.22
RBR R.1	3(2,4)	3(3,3)	2(2,3)	3(2,3)	*p* > 0.99	*p* = 0.73	*p* > 0.99
RBR R.2	4(3,4)	4(3.8,4)	3(2,3)	4(3,4)	*p* > 0.99	*p* > 0.99	*p* = 0.06
LCC R.1	3(2,4)	3(3,3)	2(2,3)	3(2,3)	*p* > 0.99	*p* = 0.73	*p* > 0.99
LCC R.2	4(3,4)	4(3.8,4)	3(2,3)	4(3,4)	*p* > 0.99	*p* > 0.99	*p* = 0.03*
LSC R.1	3(2,4)	3(3,3)	2(2,3)	3(2,3)	*p* > 0.99	*p* = 0.73	*p* > 0.99
LSC R.2	4(3,4)	4(3.8,4)	3(2,3)	4(3,4)	*p* > 0.99	*p* > 0.99	*p* = 0.02*
SVC R.1	4(3, 4.3)	4.5(3.8, 5)	3(2.8,4)	4(3,5)	*p* > 0.99	*p* > 0.99	*p* = 0.2
SVC R.2	4(3,4)	5(4,5)	4(3, 4.25)	4(3,5)	*p* > 0.99	*p* = 0.3	*p* > 0.99

* *p* < 0.05

In particular, comparison between the fully-sampled MTC-BOOST and the proposed 5-fold undersampled jMS-VNN MTC-BOOST showed similar results for both reviewers for all structures. Comparison between the proposed 5-fold jMS-VNN MTC-BOOST and the clinical reference showed similar image quality scores for both reviewers for all structures except the pulmonary veins, whereas the proposed 5-fold jMS-VNN outperformed the clinical sequence. The 5-fold jMS-VNN was superior to CS in the depiction of the coronary arteries, and ascending aorta and comparable for the rest of the structures.

In cases where the anatomy could not be fully elucidated with the clinical 3D-WH, previous imaging was utilized to evaluate the MTC-BOOST findings. The anatomy was confirmed in CT in five patients, in previous contrast-enhanced MR angiography in eleven and in previous invasive catheter procedures in two.

### 3.3 Luminal SNR for Bright-Blood jMS-VNN MTC-BOOST

SNR comparison for the 5-fold jMS-VNN and fully-sampled MTC-BOOST demonstrated no statistically significant difference in all the structures assessed.

SNR was comparable between the clinical versus the FS MTC-BOOST and 5-fold jMS-VNN for all structures except the head and neck vessels and the great arteries, where the conventional sequence was inferior to both.

Detailed results are shown in Table 4 and SM Table 3.

**Table 4:** Inter-method comparison of bright-blood signal to noise ratio for vascular structures acquired with 5-fold undersampling with jMS-VNN reconstructions versus the clinical T2prep-bSSFP, MTC-BOOST fully sampled and 5-fold undersampled with CS reconstruction.

Structure	Clinical	MTC-BOOST Fully sampled	MTC BOOST Compressed Sensing	MTC BOOST jMS-VNN	Clinical vs MTC-BOOST jMS-VNN	MTC-BOOST Fully sampled vs MTC-BOOST jMS-VNN	MTC-BOOST Compressed Sensing vs MTC-BOOST jMS-VNN
LAD	60 (52, 69)	56 (45,75)	59 (30, 65)	60 (46, 74)	*p* > 0.99	*p* > 0.99	*p* > 0.99
LCx	45 (25, 62)	37 (0, 54)	0 (0, 49)	45 (25, 62)	*p* > 0.99	*p* > 0.99	*p* > 0.99
RCA	54 (28, 83)	64 (53, 82)	74 (53, 96)	72 (63, 94)	*p* = 0.06	*p* > 0.99	*p* > 0.99
RPA	69 (55 77)	73 (|57, 89)	83 (57, 96)	91 (71, 101)	*p* > 0.99	*p* > 0.99	*p* > 0.99
LPA	67 (62, 85)	80 (61, 96)	87 (80, 103)	84 (73, 106)	*p* = 0.3	*p* > 0.99	*p* > 0.99
MPA	69 (53, 78)	87 (87, 121)	127 (92, 144)	123 (76, 134)	*p* = 0.01*	*p* > 0.99	*p* = 0.9
RPV	27 (19, 39)	29 (23, 38)	36 (33, 47)	32 (27, 42)	*p* = 0.7	*p* > 0.99	*p* > 0.99
LPV	38 (22, 54)	54 (43, 71)	63 (49. 76)	63 (49, 74)	*p* = 0.052	*p* > 0.99	*p* > 0.99
RBR	64 (56, 75)	104 (77, 132)	99 (87, 127)	107 (80, 130)	*p* = 0.005*	*p* > 0.99	*p* > 0.99
LCC	69 (60, 92)	108 (86, 129)	104 (86, 119)	107 (78, 114)	*p* = 0.03*	*p* > 0.99	*p* > 0.99
LSC	64 (31, 76)	93 (65, 107)	88 (75, 105)	93 (76, 108)	*p* = 0.02*	*p* > 0.99	*p* > 0.99
SVC	94 (70, 104)	82 (60, 94)	51 (41, 58)	73 (60, 98)	*p* > 0.99	*p* > 0.99	*p* = 0.02*

* *p* < 0.05

### 3.4 Image Quality Metrics for Bright-Blood jMS-VNN MTC-BOOST

A comparison of image quality metrics (PSNR and SSIM) for the 5-fold accelerated MTC-BOOST acquisition with CS and jMS-VNN reconstruction to the fully-sampled MTC-BOOST images demonstrated higher performance of jMS-VNN than CS, although not statistically significant (Table 5).

**Table 5:** Image quality comparison using peak signal-to-noise ratio (PSNR) and structural similarity (SSIM) over the region of interest around the heart and the thoracic vessels of the 5-fold accelerated CS and proposed 5-fold accelerated jMS-VNN.

Structures	jMS-VNN	CS
PSNR	47.39 ± 2.5 *p* = 0.33	46.8 ± 2.4
SSIM	0.72 ± 0.1, *p* = 0.37	0.69 ± 0.1

Fully sampled images were used as reference in both cases.

### 3.5 Co-axial Diameter Measurements for Bright-Blood jMS-VNN MTC-BOOST

**Fig. 7:**
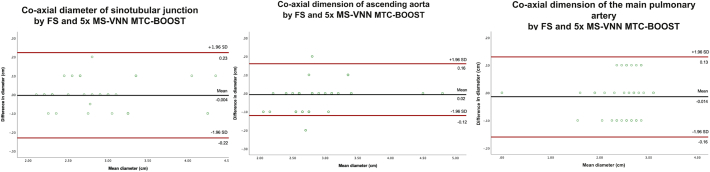
Bland-Altman plots comparing vascular dimensions between the fully-sampled MTC-BOOST and 5-fold jMS-VNN. Bland–Altman analysis comparing the aortic dimensions at the level of sinotubular junction, ascending aorta and main pulmonary artery. Measurements were performed in the 5-fold accelerated acquisition with joint Multi Scale Variational Neural Network (jMS-VNN) reconstruction and Fully Sampled MTC BOOST. The black line indicates the mean bias of the diameter measurements whereas the red lines represent the 95% confidence interval. Values are given in cm. **A:** co-axial aortic dimensions at the level of sinotubular junction demonstrate excellent agreement with a mean difference of −0.004 cm (95% confidence interval − 0.22. to 0.23, *p* value 0.7). **B:** co-axial dimensions of mid ascending aorta result demonstrate excellent agreement with a mean difference of 0.02 cm (95% confidence interval − 0.12 to 0.16, p value 0.09). **C:** co-axial dimensions of main pulmonary artery diameter demonstrate excellent agreement with a mean difference of −0.014 cm (95% confidence interval − 0.16 to 0.13, p value: 0.3). Abbreviations: FS: Fully-sampled, jMS-VNN: joint Multi Scale Variational Neural Network, MTC-BOOST: Magnetisation Transfer Contrast Bright and black blOOd phase SensiTive. (For interpretation of the references to colour in this figure legend, the reader is referred to the web version of this article.)

Co-axial diameter measurements using fully-sampled and 5-fold jMS-VNN MTC-BOOST were compared using Bland-Altman analysis for the sinotubular junction (STJ), the mid ascending aorta and the main pulmonary artery (Fig. 6). The results showed good agreement with a mean difference of <0.02 cm and narrow limits of agreement for all three structures (Fig. 7).

## 4 Discussion

In this study, an efficient deep-learning reconstruction framework for accelerated free-breathing MTC–BOOST sequence is proposed, for bright-blood contrast-free imaging in CHD. The results presented in this work show that jMS-VNN reconstruction could offer significant advantages for integration in clinical workflow: 1) it provides good quality bright blood images, comparable to the fully-sampled MTC-BOOST acquisition and comparable or superior to the clinical sequence, 2) acquisition of the 5-fold accelerated jMS-VNN dataset is achieved in approximately 3 min, enabling fast and predictable scan time and reducing the susceptibility to cardiac motion and respiratory drift, 3) the proposed framework does not require further optimisation of reconstruction parameters and achieves extremely fast computational times, retrieving fine anatomical details and yielding image quality improvement in comparison to CS in significantly faster reconstruction time (20 s). In contrary to CS methods, that perform an iterative optimization for each subject and are not able to exploit the prior knowledge that can be learned from numerous previously performed MRI acquisitions; the proposed jMS-VNN employs previously acquired data sets to learn the key reconstruction parameters and priors during an up-front training procedure, incorporating the optimization procedure in the training and rapidly reducing reconstruction time.

The proposed framework combines previously introduced translational respiratory motion correction with iNAVs, allowing for nearly 100% scan efficiency and predictable scan time and an undersampled variable density spiral-like Cartesian trajectory, which introduces incoherent noise-like artefacts, enabling high undersampling factors.

The 5-fold jMS-VNN and fully-sampled MTC-BOOST resulted in improved SNR in the great arteries, which can be possibly attributed to the attenuation of flow-related artefacts due to the MT preparation pulse. MT preparation, contrary to the T2-prep bSSFP, has been previously shown to be robust in vascular imaging, even in areas with fast flowing blood [26]. In our study, this is only reflected in statistically significant difference in the SNR and not in the image quality scores, possible due to the size of the cohort and the underlying diagnoses. High frequency offset (3000 Hz) was chosen for MT preparation to minimize image artefacts in regions with imperfect B0 and shimming and to minimize on-resonance saturation, which has also improved the SNR in the neck vessels. Furthermore, the noise penalty induced by the g-factor maps (parallel imaging) in the clinical 3D-WH, has been shown to result in higher noise level [27]. PSNR and SSIM quantitative metrics demonstrated higher performance of jMS-VNN than CS, although not statistically significant.

The use of sensitivity encoding (SENSE) [28] has been shown to reduce scan time in 3D-WH imaging in CHD. However, the application of deep-learning reconstruction methods for 3D-WH imaging for CHD patients has limited description in the literature [29]. Steeden et al. [29] has recently demonstrated the potential of a deep-learning based super resolution reconstruction of rapidly sampled low-resolution T2prep-3D-WH images using a residual U-Net. This approach was evaluated in patients with CHD and operates on coil-combined low-resolution single-contrast images acquired with diaphragmatic navigators. The principal limitation of this technique lies in that the validation data consist of coil-combined magnitude images, instead of multi-coil k-space data. On the contrary, unrolled deep-learning methods such as VNN [20], are trained in order to reconstruct the MR images directly from the undersampled k-space data as an input, thus incorporating the known physics model of the MR acquisition into the training and result in a data-driven and model-based framework for undersampled MR reconstruction. Additionally, our approach operates on 5-fold undersampled two-contrast k-space data with translational respiratory motion correction through image based navigators (100% respiratory scan efficiency, i.e. data acquired across the entire respiratory cycle is used for reconstruction, no data rejection).

Further applications of deep-learning based reconstruction in cardiac MRI have been proposed [[30], [31], [32], [33], [34], [35], [36]], including 4D CINEnet for cartesian undersampled 3D isotropic cine imaging [35], deep-cascade of convolutional neural networks for cartesian single-coil undersampled 2D cardiac images [37] and convolutional recurrent neural networks for dynamic MRI reconstruction that exploit the dependencies of the temporal sequences and the iterative nature of the traditional optimisation algorithms [38]. However, none of these is implemented for respiratory motion-compensated 3D whole-heart imaging in CHD. Several studies have demonstrated that the performance of deep-learning based approaches in MRI image reconstruction is comparable or superior to conventional CS-based methods [35,[39], [40]]. Some studies compared the deep-learning based reconstructed datasets with the CS datasets, serving as practical ground truth data; as it was not possible to have fully-sampled ground-truth data [39-40]. In our study we examined the performance of the proposed network against the clinical standard, the fully-sampled MTC-BOOST and 5-fold accelerated with CS.

MS-VNN has been previously proposed to accelerate single-contrast, free-breathing, motion-compensated, isotropic, high-resolution whole heart 3D Coronary Magnetic Resonance Angiography in healthy subjects, presenting improved image quality when compared to zero-filled and CS reconstructions [20]. Here this approach has been extended to enable simultaneous reconstruction of two contrasts (MTC-IR and MTC), providing bright and PSIR black blood MTC-BOOST images in ~20s reconstruction time and has been evaluated in a cohort of 18 CHD patients with a range of conditions.

This work has some limitations. In the present study the scan time for the fully-sampled MTC-BOOST sequence is lower compared to the clinical sequence, but the acquired resolution in the z-direction is half compared to the bright-blood clinical sequence. Isotropic acquisition will be investigated in future work. Respiratory motion correction was performed beat-to-beat for 2D translational motion only; non-rigid motion correction should be considered in future studies to account for the complex motion of the heart [41]. In addition, arrythmia rejection was not included in the acquisition/reconstruction and although there was no issue in the observed cohort, it requires further investigation. This is a proof of concept study to investigate the quality of the 5-fold accelerated bright-blood MTC-BOOST for the visualization of cardiac and vascular anatomy in CHD patients compared to a standard non contrast-enhanced bright-blood T2prep-3DWH imaging technique. There is supporting evidence that PSIR (black-blood dataset) carries important diagnostic information for thrombus visualization that is relevant for Fontan and baffle (e.g. Mustard procedure) assessment. However, this has not been fully elucidated with the fully-sampled black-blood MTC-BOOST dataset and therefore was not investigated in the 5-fold accelerated either. Future studies should further investigate the clinical value of the PSIR black-blood MTC-BOOST volume in the CHD population in comparison to conventional black-blood techniques. The jMS-VNN reconstruction was implemented offline. Whilst this may be suitable for initial testing, the inline integration of this technique will be key for adoption in clinical practice. This is a single-center study recruiting a relatively small cohort of adult patients with CHD. This should be extended in future studies to incorporate a larger cohort and paediatric patients from additional centres and thus to investigate extensive application of this technique and its direct clinical implications with regards to its capability to preserve unique pathologies in patients with different somatometrics, underlying anatomy and heart rate variability.

## 5 Conclusion

We propose an efficient reconstruction framework for free-breathing 3D whole-heart imaging that combines a prototype MTC-BOOST sequence, translational respiratory motion correction with iNAVs, and a variable density spiral-like Cartesian trajectory with a novel joint Multi-Scale Variational Neural Network reconstruction, for 5-fold accelerated acquisition and extremely fast reconstruction. The proposed approach outperforms traditional CS and is comparable to fully-sampled the MTC-BOOST and conventional bright-blood T2-prepared bSSFP 3D whole-heart imaging in an anatomically diverse population. This along with short acquisition and reconstruction time is promising potential clinical adoption.

The following are the supplementary data related to this article.

Supplementary Figure 1 Black-blood images in coronal view, for five representative participants. Acquisitions were performed with the fully sampled MTC-BOOST sequence and 5x prospectively undersampled MTC-BOOST sequence. Accelerated MTC-BOOST was reconstructed with CS and jMS-VNN. CS reconstruction introduces residual blurring in the delineation of left ventricular wall and papillary muscles (yellow box), right ventricular trabeculations (blue box) and left ventricular wall and papillary muscles (red box), (Participant 2,8,15 respectively). jMS-VNN shows higher image quality than CS, achieving similar image quality to the fully-sampled scan. Congenital Heart Disease (CHD), Compressed Sensing (CS), Joint Multi Scale Variational Neural Network (jMS-VNN), Magnetisation Transfer Contrast Bright and black blOOd phase SensiTive (MTC-BOOST). Supplementary Figure 1

Supplementary material 1 Image 1

## Funding Sources

The authors acknowledge financial support from the 10.13039/501100000274BHF PG/18/59/33955, 10.13039/501100000266EPSRC EP/P001009, EP/P032311/1, EP/P007619, Wellcome EPSRC Centre for Medical Engineering (NS/A000049/1), and the 10.13039/501100000272Department of health via the National Institute for Health Research (NIHR) comprehensive 10.13039/100014461Biomedical Research Centre award to Guy's and St. Thomas' NHS Foundation Trust.

## Ethics Approval

All procedures performed in this study were in accordance with the 1964 Declaration of Helsinki and its later amendments or comparable ethical standards. The study was approved by the National Research Ethics Service (REC 15/NS/0030).

## Informed Consent

Written informed consent was obtained from each participant ac- cording to institutional guidelines.

## Data Statement

Data generated or analysed during the study are available from the corresponding author upon reasonable request.

## Declaration of Competing Interest

All the authors declare that they do not have competing interests.
