# Supplementary material for: Accelerating 3D MTC-BOOST in patients with congenital heart disease using a joint multi-scale variational neural network reconstruction
Source: Magn Reson Imaging. 2022 Oct;92:120–32. doi: 10.1016/j.mri.2022.06.012 (PMC9826869; doi:10.1016/j.mri.2022.06.012)
Supplement: Supplementary Figure 1 — Black-blood images in coronal view, for five representative participants. Acquisitions were performed with the fully sampled MTC-BOOST sequence and 5x prospectively undersampled MTC-BOOST sequence. Accelerated MTC-BOOST was reconstructed with CS and jMS-VNN. CS reconstruction introduces residual blurring in the delineation of left ventricular wall and papillary muscles (yellow box), right ventricular trabeculations (blue box) and left ventricular wall and papillary muscles (red box), (Participant 2,8,15 respectively). jMS-VNN shows higher image quality than CS, achieving similar image quality to the fully-sampled scan. Congenital Heart Disease (CHD), Compressed Sensing (CS), Joint Multi Scale Variational Neural Network (jMS-VNN), Magnetisation Transfer Contrast Bright and black blOOd phase SensiTive (MTC-BOOST). [file mmc1.zip › mmc1.pptx]

## Slide 1
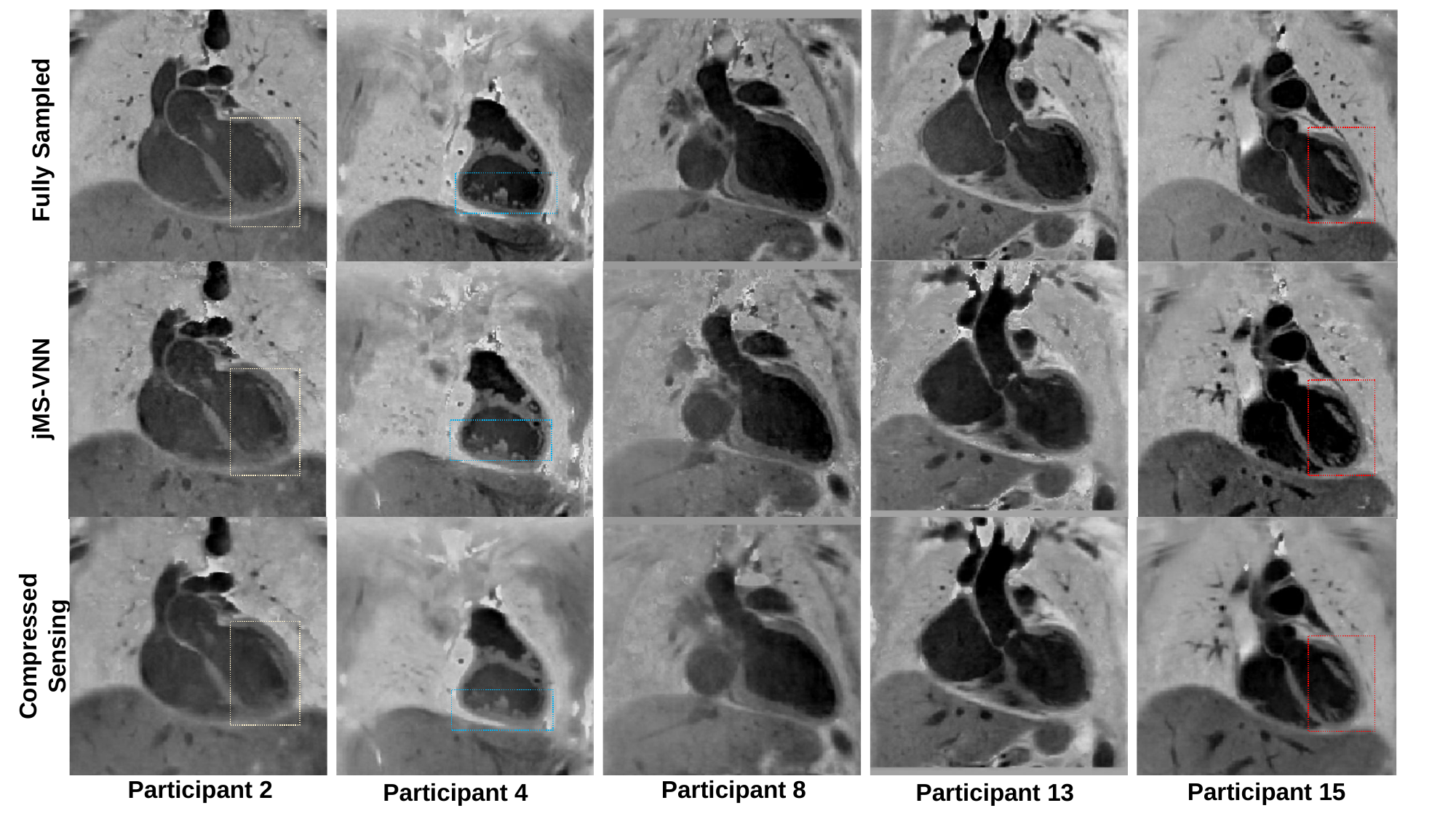

Fully Sampled
jMS-VNN
Compressed Sensing
Participant 2
Participant 8
Participant 15
Participant 4
Participant 13
