# Supplementary material for: Accelerating 3D MTC-BOOST in patients with congenital heart disease using a joint multi-scale variational neural network reconstruction
Source: Magn Reson Imaging. 2022 Oct;92:120–32. doi: 10.1016/j.mri.2022.06.012 (PMC9826869; doi:10.1016/j.mri.2022.06.012)
Supplement: Supplementary file 2 — Supplementary material 1 [file mmc2.docx]

**Supplementary Material Table 1**: Patient identification number (ID), age, corresponding diagnosis and procedures for the participants included in the training of the proposed jMS-VNN network.

| Patient ID | Age (years) | Diagnosis | Procedures |
| --- | --- | --- | --- |
| 1 | 25 | Double outlet right ventricle  Mild main pulmonary artery stenosis | 1.Right modified Blalock-Taussig shunt  2.Double outlet right ventricle repair  3.Balloon angioplasty of the right pulmonary artery |
| 2 | 24 | Dextrocardia  Left atrial isomerism  Atrioventricular septal defect | 1.Repair of atrioventricular septal defect |
| 3 | 24 | Hypoplastic left heart syndrome | 1.3-staged Norwood operation  2.Left pulmonary artery stent |
| 4 | 20 | Scimitar syndrome  Severe right pulmonary artery hypoplasia  Supra-valvar mitral membrane | Supra-valvar mitral membrane resection |
| 5 | 23 | Double outlet right ventricle | 1.Pulmonary artery banding and atrial septectomy  2.Arterial switch operation with ventricular and atrial septal defect closure |
| 6 | 19 | Mitral valve dysplasia  Aortopulmonary window | 1.Mitral valve repair  2.Aortopulmonary window closure |
| 7 | 35 | Coarctation of the aorta | 1.Surgical repair of coarctation with 20mm Hemashield interposition graft |
| 8 | 30 | Coarctation of the aorta  Ventricular septal defect  Subaortic stenosis | 1.Subclavian flap repair, ventricular septal defect closure and resection of subaortic stenosis |
| 9 | 21 | Double inlet left ventricle with ventriculoarterial discordance  Coarctation of the aorta  2:1 heart block  Epicardial dual chamber pacemaker | 1.Coarctation of the aorta repair and pulmonary artery banding  2.Completion of lateral tunnel Fontan |
| 10 | 34 | Coarctation of the aorta  Bicuspid aortic valve | 1.Subclavian flap repair of coarctation  2.Ross procedure. Pulmonary homograft in situ |
| 11 | 49 | Atrial septal defect  Aortic regurgitation |  |
| 12 | 18 | Bicuspid aortic valve  Aortic root dilatation |  |
| 13 | 17 | Bicuspid aortic valve |  |
| 14 | 18 | Pulmonary atresia  Atrial septal defect | 1.Radiofrequency valvotomy for pulmonary atresia, Patent ductus arteriosus stent  Atrial septal defect device closure |
| 15 | 35 | Partial anomalous pulmonary venous drainage |  |
| 16 | 33 | Pulmonary stenosis  Ventricular septal defect | 1.Ventricular septal defect patch closure  Resection of subpulmonary stenosis |
| 17 | 35 | Pulmonary stenosis  Atrial septal defect  Ventricular septal defect | 1.Surgical closure of atrial and ventricular septal defect Implantation of bioprosthetic valve in pulmonary position |
| 18 | 27 | Bicuspid aortic valve |  |

**Supplementary Material Table 2:** Inter-method comparison of bright-blood image quality scores for vascular structures acquired with the clinical T2prep-bSSFP sequence, the MTC-BOOST fully sampled and 5-fold undersampled with CS reconstruction.

| Structure | Clinical | MTC-BOOST  Fully sampled | MTC-BOOST  Compressed  Sensing | Clinical vs  MTC-BOOST  Fully sampled | Clinical vs MTC-BOOST  Compressed Sensing | MTC-BOOST  Fully sampled  vs MTC-BOOST  Compressed  Sensing |
| --- | --- | --- | --- | --- | --- | --- |
| LAD R.1 | 2(2,2) | 2(1, 2) | 1(1,1.3) | P>0.99 | P=0.0003* | P=0.01* |
| LAD R.2 | 4(3,4) | 3.5(3,4) | 2(2.3) | P>0.99 | P<0.0001* | P=0.0002* |
| LCx R.1 | 2(2,2) | 2(1, 2) | 1(1,1.3) | P=0.9 | P<0.0001* | P=0.007* |
| LCx R.2 | 4(3,4) | 3.5(3,4) | 2(2,3) | P>0.99 | P<0.0001* | P=0.002* |
| RCA R.1 | 2(2,2) | 2(1, 2) | 1(1,1.3) | P=0.9 | P<0.0001* | P=0.008* |
| RCA R.2 | 4(3,4) | 3(2,4) | 2(2.3) | P>0.99 | P<0.0001* | P<0.0001* |
| AA R.1 | 4(4,4) | 4(3,4) | 3(3,3) | P=0.7 | P=0.0006* | P=0.13 |
| AA R.2 | 4(3.8,4) | 4(3,4) | 3(3,3.5) | P>0.99 | P=0.006* | P=0.0004* |
| RPA R.1 | 4(4,4) | 4(3,4) | 3(3,3) | P=0.8 | P<0.0001* | P=0.02* |
| RPA R.2 | 4(3.8,4) | 4(3.8,4) | 3(3,3) | P>0.99 | P=0.02* | P=0.01* |
| LPA R.1 | 4(4,4) | 4(3,4) | 3(3,3) | P=0.8 | P<0.001* | P=0.03* |
| LPA R.2 | 4(3.8,4) | 4(3.8,4) | 3(3,3) | P>0.99 | P=0.02* | P=0.01* |
| MPA R.1 | 4(4,4) | 4(3,4) | 3(3,3) | P=0.8 | P<0.0001* | P=0.02* |
| MPA R.2 | 4(3.75,4) | 4(3.8,4) | 3(3,4) | P>0.99 | P=0.006* | P=0.007* |
| RPV R.1 | 1(1,2) | 3.5(2.8,4) | 3(2,3) | P<0.006* | P=0.01* | P=0.3 |
| RPV R.2 | 2(1.8,2.3) | 4(3,4) | 3(2,3) | P<0.0001* | P=0.08 | P=0.09 |
| LPV R.1 | 1(1,2.3) | 3.5(2.8,4) | 3(2,3) | P<0.0001* | P=0.03* | P=0.5 |
| LPV R.2 | 3(2,3.3) | 3.5(3,4) | 4(3,4) | P=0.0005* | P>0.99 | P=0.02* |
| RBR R.1 | 3(2,4) | 3(3,3) | 2(2,3) | P>0.99 | P=0.06 | P=0.03* |
| RBR R.2 | 4(3,4) | 4(3.8,4) | 3(2,3) | P>0.99 | P=0.1 | P=0.002* |
| LCC R.1 | 3(2,4) | 3(3,3) | 2(2,3) | P>0.99 | P=0.06 | P=0.03* |
| LCC R.2 | 4(3,4) | 4(3.8,4) | 3(2,3) | P>0.99 | P=0.1 | P=0.002* |
| LSC R.1 | 3(2,4) | 3(3,3) | 2(2,3) | P>0.99 | P=0.06 | P=0.03* |
| LSC R.2 | 4(3,4) | 4(3.8,4) | 3(2,3) | P=>0.99 | P=0.11 | P=0.006* |
| SVC R.1 | 4(3, 4.3) | 4.5(3.8, 5) | 3(2.8,4) | P=0.6 | P=0.8 | P=0.01* |
| SVC R.2 | 4(3,4) | 5(4,5) | 4(3, 4.25) | 0.02* | p>0.99 | P=0.005* |

Abbreviations: AA: Ascending aorta, CS: Compressed sensing, jMS-VNN: joint Multi Scale Variational Neural Network, LAD: Left anterior descending coronary artery, LCC: Left common carotid, LCx: Left circumflex coronary artery, LPA: Left pulmonary artery, LPV: Left pulmonary vein, LSC: Left subclavian, MPA: Main pulmonary artery, MTC-BOOST: Magnetisation Transfer Contrast Bright and black blOOd phase SensiTive, RBR: Right brachiocephalic, RCA: Right coronary artery, RPA: Right Pulmonary Artery, SVC: Superior vena cava, T2prep-bSSFP: T2prepared-balanced Steady State Free Precession.

**Supplementary Material Table 3:** Inter-method comparison of bright-blood SNR scores for vascular structures acquired with the clinical T2prep-bSSFP sequence, the MTC-BOOST fully sampled and 5-fold undersampled with CS reconstruction.

| Structure | Clinical | MTC-BOOST  Fully sampled | MTC BOOST  Compressed  Sensing | Clinical vs  MTC-BOOST  Fully sampled | Clinical vs MTC-BOOST  Compressed  Sensing | MTC-BOOST  Fully sampled  vs MTC-BOOST  Compressed  Sensing |
| --- | --- | --- | --- | --- | --- | --- |
| LAD | 60 (52, 69) | 56 (45,75) | 59 (30, 65) | P>0.99 | P>0.99 | P>0.99 |
| LCx | 45 (25, 62) | 37 (0, 54) | 0 (0, 49) | P>0.99 | P=0.1 | P>0.99 |
| RCA | 54 (28, 83) | 64 (53, 82) | 74 (53, 96) | P>0.99 | P=0.2 | P>0.99 |
| RPA | 69 (55 77) | 73 (\|57, 89) | 83 (57, 96) | P>0.99 | P=0.06 | P=0.4 |
| LPA | 67 (62, 85) | 80 (61, 96) | 87 (80, 103) | P>0.99 | P=0.1 | P=0.9 |
| MPA | 69 (53, 78) | 87 (87, 121) | 127 (92, 144) | P=0.04* | P<0.00018 | P=0.3 |
| RPV | 27 (19, 39) | 29 (23, 38) | 36 (33, 47) | P>0.99 | P=0.53 | P=0.18 |
| LPV | 38 (22, 54) | 54 (43, 71) | 63 (49. 76) | P=0.2 | P=0.06 | P>0.99 |
| RBR | 64 (56, 75) | 104 (77, 132) | 99 (87, 127) | P=0.005* | P=0.004* | P>0.99 |
| LCC | 69 (60, 92) | 108 (86, 129) | 104 (86, 119) | P=0.003* | P=0.03* | P>0.99 |
| LSC | 64 (31, 76) | 93 (65, 107) | 88 (75, 105) | P=0.01* | P=0.05* | P>0.99 |
| SVC | 94 (70, 104) | 82 (60, 94) | 51 (41, 58) | P>0.99 | P=0.0004* | P=0.01* |

Abbreviations: AA: Ascending aorta, CS: Compressed sensing, jMS-VNN: joint Multi Scale Variational Neural Network, LAD: Left anterior descending coronary artery, LCC: Left common carotid, LCx: Left circumflex coronary artery, LPA: Left pulmonary artery, LPV: Left pulmonary vein, LSC: Left subclavian, MPA: Main pulmonary artery, MTC-BOOST: Magnetisation Transfer Contrast Bright and black blOOd phase SensiTive, RBR: Right brachiocephalic, RCA: Right coronary artery, RPA: Right Pulmonary Artery, SNR: Signal to Noise Ratio, SVC: Superior vena cava, T2prep-bSSFP: T2prepared-balanced Steady State Free Precession.
